# Addressing systemic workforce challenges in general practice—a qualitative study of general practitioners in Ireland

**DOI:** 10.1093/fampra/cmaf094

**Published:** 2025-12-09

**Authors:** Uzair Shabbir, Ray O’Connor, Joe MacDonagh, Andrew O’Regan

**Affiliations:** School of Medicine, University of Limerick, Limerick V94 T9PX, Ireland; Health Research Institute, University of Limerick, Limerick V94 T9PX, Ireland; School of Medicine, University of Limerick, Limerick V94 T9PX, Ireland; Strategy & Leadership Discipline, Faculty of Business, Technological University Dublin, Dublin D07 EWV4, Ireland; School of Medicine, University of Limerick, Limerick V94 T9PX, Ireland; Health Research Institute, University of Limerick, Limerick V94 T9PX, Ireland

**Keywords:** general practice, workforce planning, health services accessibility, health systems/policy, qualitative research

## Abstract

**Background:**

General practice across Europe faces a workforce crisis, with a projected shortfall of up to 1660 general practitioners in Ireland by 2028. While policy interventions have been proposed, a gap remains between the Irish health system's strategic objectives and the day-to-day realities experienced by general practitioners.

**Objective:**

The aim of this study is to explore the perspectives of Irish general practitioners in addressing recruitment and retention challenges in general practice. Specific objectives include identifying solutions and the supports necessary for sustainable future general practice.

**Methods:**

A qualitative study design was employed, utilizing semi-structured online interviews with general practitioners recruited through a network affiliated with a university. Thematic analysis was conducted by four experienced researchers. Data collection continued until thematic saturation was achieved.

**Results:**

Three primary themes emerged: (i) Towards a More Effective Health Service—participants emphasized the necessity for a whole-system approach to address recruitment and retention shortfalls; (ii) Role Clarification, Boundary Setting, and Support—participants highlighted the need for role reallocation within multidisciplinary teams to allow them to focus on complex cases; and (iii) Practice-Level Response—digital infrastructure improvements and administrative task reallocation were identified as key strategies to reduce workload and enhance patient care.

**Conclusion:**

To address the general practice workforce crisis, systemic reforms, expanded multidisciplinary teams, and practice-level adaptations are needed. The findings reflect the importance of general practitioner involvement in healthcare planning and policy development. These insights will inform targeted policy interventions in Ireland and in healthcare systems facing similar workforce challenges.

Key messages

- Systemic reforms in the wider Irish health system are essential for sustainable recruitment and retention in general practice.
- Clarifying multidisciplinary team roles can reduce workload and improve patient care.
- Using effective computer systems and applications can reduce administrative burdens and support general practitioners in focusing on patient care.
- General practitioners' involvement in healthcare planning optimizes general practice performance by highlighting system failings and proposing solutions.
- Rural general practice has specific needs and challenges that are different to urban general practices.

## Introduction

Across Europe, general practice faces immense strain due to workforce shortages, compounded by an ageing workforce, rising administrative burdens, and increasing patient care demands, which together exacerbate recruitment and retention challenges, with general practitioners retiring at a younger age, not staying on past retirement as they did traditionally and choosing to emigrate [1–6]. In Ireland, the situation is particularly serious, with an estimated 29 million consultations annually amidst severe workforce shortages and escalating demands on general practitioners (GPs) [7]. By 2028, a workforce deficit of 1260–1660 GPs is projected, leaving many practices, particularly in rural areas, unable to accept new patients [3, 8]. GPs work extended hours, often up to 10 hours per day, yet growing administrative burdens detract from direct patient care [9].

Recently, the Economic and Social Research Institute (ESRI) reported on the general practice workforce in Ireland, anticipating that the demand for GP consultations will rise by 23–30% between 2030 and 2040 [10]; according to the report, the main factors driving the projected increase in demand are increasing population size and an ageing population. Also in 2025, the Irish College of General Practitioners (ICGP) published “Strengthening the Future of GP Care in Ireland [11]. Similarly, rising numbers and ageing of the population are identified as key factors contributing to the strain on GPs as well as the relatively low GP to population ratios: Ireland has 60 GPs per 100 000 people, which is well below neighbouring countries; the UK for instance has a GP to population ratio of 100 per 100,000 [11]. The problem is further complicated by significant numbers of Irish-trained GPs emigrating each year to countries such as Australia, New Zealand, Canada, and the UK [12]. The implications of this lack of GP personnel in the workforce are further increased workload for GPs and consequently more burnout and early retirement; further strain on the healthcare system and difficulties with access to healthcare for patients who are unable to get appointments with their GP or to register with a GP in the first instance [13, 14].

Despite interventions proposed by the ICGP, such as workforce expansion and improved infrastructure [3, 15], few studies have examined the in-depth lived experiences of GPs working in such an embattled system [16, 17]. Addressing calls for deeper insights into GP experiences [4, 16, 17], this research complements existing quantitative findings by providing qualitative insights into the daily challenges, needs, and proposed solutions of GPs [3, 5, 15, 18].

The aim of this study is to investigate GP perspectives on how to address recruitment and retention challenges in Ireland effectively. Specific objectives are to:

- Explore solutions from the experience of GPs working in the Irish healthcare system
- Identify the changes GPs believe are necessary to ensure that general practice is sustainable.

## Methods

### Setting

This study was conducted in the Irish general practice setting [19]. Ireland operates a mixed public/private healthcare system—as of 2024, 29.7% of the population held a General Medical Services (GMS) medical card [20, 21], which provides free general practice care for individuals based on financial need and age [22]. An additional 12.3% of the population qualified for a GP visit card, granting limited free general practice services, including for those over 70 years and children under 8 years [20, 21]. The remaining 58% of the population are private patients, a proportion of whom lack both medical cards and private health insurance [20]. Some GMS patients also hold private health insurance, reflecting the nuanced dynamics of Ireland's healthcare system, where individuals may rely on multiple coverage types to access care [20].

### Study design and participants

This qualitative study utilized semi-structured online interviews with practising GPs. Ethical approval was obtained from the Education and Health Sciences Research Ethics Committee of the University of Limerick (ID: 2023_03_12_EHS). Informed consent was obtained from all participants following the distribution of an information sheet detailing the study objectives, researcher roles, and any potential risks and benefits of participation. The study methodology adhered to the Consolidated Criteria for Reporting Qualitative Research (COREQ) [23].

Participants were recruited via an online invitation to the University of Limerick Education and Research Network for General Practitioners (ULEARN-GP), which reflects national GP demographics in age, gender, and rural-urban distribution [24]. The convenience non-probabilistic sample included 21 respondents (see Table 1). For qualitative studies, a self-selected sample is acceptable, in that we are exploring the workplace challenges Irish GPs faced, rather than seeking to claim that all Irish GPs faced these equally, or that these were the only challenges faced by Irish doctors in general practice-as might be the case in a random sample from a representative sampling frame, with a research question examining the specific challenges facing Irish GPs. The approach was to interview an adequate number of GPs to identify the depth and nature of their experience of the challenges they faced, rather than seeking to identify challenges representative of all GPs' experience.

**Table 1.** Participant demographics and professional background.

Gender	Women (*n* = 6)	Men (*n* = 15)		
Age	30–39 (*n* = 6)	40–49 (*n* = 5)	50–59 (*n* = 2)	60 and above (*n* = 8)
Role in the practice	Partner (*n* = 15)	Salaried (*n* = 4)	Retired (*n* = 2)	
Years working as a GP	1–9 (*n* = 8)	10–19 (*n* = 3)	20–29 (*n* = 2)	30 + years (*n* = 8)

This table provides an overview of the demographics and professional roles of the 21 study participants, including gender, age, years of experience, and additional roles within general practice in Ireland.

### Data collection and management

The development of the semi-structured interview guide was shaped by preliminary discussions with experts on qualitative methods and general practice research. Additionally, insights from a preliminary focus group comprising representative GPs guided the direction of this study (the findings of the focus group were used to guide the study only and were not included in the analysis). The focus group was designed using input from an expert on participatory health research.

Interviews were conducted (by U.S.) between January 2023 and August 2023 using Microsoft Teams and were between 20 and 60 minutes, with an average duration of 47 minutes. Interviews were digitally recorded, and comprehensive field notes were taken concurrently. The recordings were transcribed verbatim and anonymized by removing identifiers. The interview guide was dynamically refined in response to emerging themes from the data, allowing for deeper exploration of themes in subsequent interviews. Data collection continued until saturation was reached.

### Data analysis

A team of four experienced qualitative researchers from the fields of general practice and business and organizational psychology (U.S., R.O.C., J.M.D., and A.O.R.) conducted the data analysis using the principles of inductive thematic analysis, as outlined by Braun and Clarke—the developers and main proponents of inductive thematic analysis [25]. Braun and Clarke suggest a minimum sample size of six for thematic analysis, in order for major and minor themes to emerge and to attain data saturation, i.e. when the themes start to be repeated in the sample by new participants [26]. The present study had a sample of 21, and it was decided to analyse all of those who had agreed to participate so that it could be seen how the themes emerged across all participants. Thus, the sample size exceeded both the accepted thematic analysis minimum sample size and allowed for the themes of all demographic groups in the sample to be represented [27]. It was clear by the 18th transcript that no new perspectives or opinions were emerging.

The audio recordings and transcripts were reviewed to gain familiarity with the data. Each researcher then independently analysed the transcripts to identify emergent codes and themes. The team met online to review and refine these themes, enhancing analytic rigour and minimizing potential bias by testing the suggested themes through collective discussion. This step-by-step collaborative approach improved analytical rigour and reflexiveness of the researchers [28]. The data analysis produced suggestions from participants as to how the Irish health system could be improved.

Key findings were organized by grouping related themes into overarching categories. This methodology provided a comprehensive exploration and understanding of the experiences and perspectives of GPs.

## Results

### Participant characteristics

Twenty-one GPs participated, reflecting a range of experience levels (see Table 1 for demographics). Most were in partner roles with over ten years of experience.

**Figure 1.**
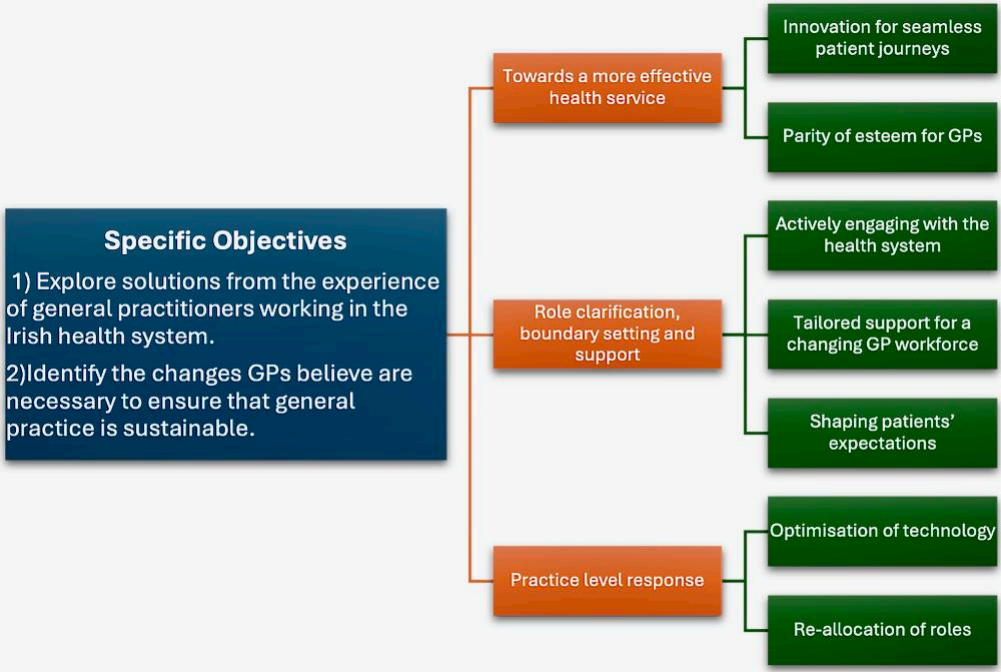
Diagrammatic representation of the themes. This figure presents the study's objectives and the proposed solutions from GPs, categorized into themes for enhancing health services, supporting GP roles, and implementing practical changes within practices.

The study identified three core themes related to the research question: Towards a More Effective Health Service; Role Clarification, Boundary Setting and Support; and Practice-Level Response, as shown in Fig. 1.

### Major theme 1—Towards a more effective health service

GPs voiced a shared commitment to the healthcare of their patients. However, the consensus among the participants was clear: the current system is inadequate, with serious consequences for patient care. GPs highlighted a range of systemic issues, such as overcrowded emergency departments, unsafe delays in outpatient and radiology appointments, fragmented communication between primary and secondary care, and insufficient access to community-based services such as physiotherapy and mental health support. These gaps in service delivery directly affect general practice, ultimately undermining continuity and quality of care.

Two interwoven subthemes emerged within this broader theme: Innovation for Seamless Patient Journeys, and Parity of Esteem for GPs—each pointing to the essential changes needed in patient care within general practice.

#### Innovation for seamless patient journeys

This subtheme centres on enhancing the interface between general practice and hospitals to create more integrated patient care pathways. Participants stated how improved technological integration could facilitate GPs with real-time access to essential patient information, including blood test results and electronic discharge summaries. These improvements would provide GPs with timely updates on patient status and treatment plans, including medication adjustments, to ensure continuity of care from hospital to home.

Currently, gaps in this interface often delay or complicate follow-up care. One GP commented:

*“We’re not integrated with hospitals in terms of IT, and that leaves us completely separate. Even when we do get discharge letters, some are still handwritten” (Interview 4, Female GP, 30–39).*

Another participant highlighted how real-time access to hospital blood tests could have an immediate impact on decision-making in primary care, reducing delays, and supporting accurate, prompt interventions.

#### Parity of esteem for GPs

GPs often voiced frustration at being treated as peripheral within the healthcare system, frequently tasked with follow-up responsibilities better suited for hospital care—a practice they described as “hospital dumping.” This occurs when hospitals discharge patients with instructions for GPs to manage follow-ups or handle complex post-discharge care without sufficient support. One GP remarked:

*“Hospital dumpage is a real issue. We’re expected to take over tasks that aren’t our responsibility, but if anything goes wrong, it's on us” (Interview 9, Male, 40–49).*

This practice not only adds to GPs' workloads but also raises medico-legal concerns, as GPs may lack the resources necessary for effective follow-up care.

While some hospitals have committees for GP–hospital integration, GPs reported that these structures rarely lead to practical improvements, further contributing to the perception among GPs that their roles and contributions are undervalued within the larger healthcare system. GPs emphasized the need for a clear delineation of responsibilities between hospitals and primary care, as well as a systemic acknowledgement of GPs' unique contributions. Participants believed that substantial attitudinal shifts are needed relating to how GPs are valued and supported.

### Major theme 2—Role clarification, boundary setting, and support

Building on the need for a more integrated healthcare system, GPs stressed the importance of clearer roles, defined boundaries, and dedicated support within general practice. A common concern was the lack of recognition by policymakers and managers of the diverse roles that GPs play and the pressures they face. While the older and more experienced participants tended to play a traditional full-time partnership role, younger GPs increasingly seek structured and flexible career paths with clear role definitions and support in their career development.

This theme addresses how general practice roles can be better understood, including activities that reduce overlap with hospital functions, and support that is appropriate to the career stage. Three subthemes emerged: Actively Engaging with the Health System; Tailored Support for a Changing GP Workforce, and Shaping Patients' Expectations.

#### Actively engaging with the health system

This subtheme highlights the need for region-specific advocacy and a collaborative approach to policy development, allowing GPs to shape solutions that meet the distinct needs of their communities. Participants stressed that effective healthcare solutions must address local and regional contexts rather than relying on a “one size fits all” approach. Many GPs expressed frustration that often policymakers lack a genuine understanding of general practice:

“They have very little knowledge of or interest in how general practice works. They really have never got inside the head of a GP” (Interview 18, Male GP, 60+).

GPs pointed out the varied challenges faced by urban versus rural areas, where demographic needs, doctor availability, and access to specialists differ significantly. A specific concern that was voiced among GPs in partnership roles was the recent expansion of medical card entitlements, which increases workload while reducing private income for GPs, often implemented without adequate consultation. Notably, participants in part-time and more portfolio careers did not reference this as an important factor. Reflecting on the policy of universal free healthcare in this context, a rural GP described the initiative as unrealistic:

*“Pure madness. I mean, you've got a 50-seater bus and now you're trying to put 100 people on it and still have a safety… People who do not have to pay for a service use the services three to four times more frequently than the private patient… The crisis at the moment is so bad that the ship of general practice is taking water” (Interview 3, Male GP, 60+).*

#### Tailored support for a changing general practitioner workforce

The traditional general practice contract no longer aligns with the evolving life and career expectations of younger GPs, who often seek flexibility, work-life balance, and diverse career opportunities. Participants widely agreed that attracting and retaining new GPs requires both financial and structural changes within general practice.

One promising approach discussed was a “trial period” scheme, allowing newly trained GPs to rotate through various practices for short-term placements (6–12 months). This model would enable young GPs to explore different communities without the pressure of immediate, long-term commitments, potentially leading to permanent placements in rural settings. Another GP suggested incentives like those in Australia, where rural service is rewarded with points towards future urban job applications, making rural work both attractive and beneficial for career progression. Additionally, purpose-built medical centres with integrated services were proposed to draw new GPs to rural areas, as younger practitioners often prefer the infrastructure of urban settings:

“*You wouldn’t expect a teacher to build their own school, yet we expect GPs… to borrow substantially to set up what is effectively a community service” (Interview 3, Male GP, 60+)​*.

Participants also emphasized the importance of a reliable locum system to support GPs' taking holidays and family-related leave. Proposed solutions included a government-subsidized locum pool to provide GPs with the flexibility to take time off without compromising patient care or their own financial stability. While a locum system could ease workloads and improve mental well-being, participants noted that careful integration is necessary to maintain continuity of patient care.

Finally, reform of out-of-hours services was highlighted as essential to reduce burnout and enhance recruitment, particularly among younger GPs, who were less interested in working unsociable hours. However, implementing these changes would require increased resources from the government to be effective.

#### Shaping patients' expectations

In line with the need for role clarity and structured support within general practice, participants emphasized the importance of reshaping patient expectations to foster a sustainable healthcare model. Many called for a structured public awareness campaign to educate patients on appropriate healthcare use, self-care, and the evolving role of general practice.

*“I would love to see a public awareness campaign to roll out and explain general practice. You know, the new model that makes patients aware of the fact that it no longer is that I have to see the doctor for my healthcare” (Interview 5, Female GP, 30–39)​.*

Such initiatives could help alleviate pressures on GPs by guiding patients towards self-management of minor illnesses and reducing unnecessary appointments.

Participants highlighted the need to empower individuals to make informed health decisions, describing a “revolving door system” in which patients frequently visit for minor, self-limiting conditions, such as colds or viral infections. GPs pointed to the success of public health messaging during the COVID-19 pandemic as evidence that a coordinated, multi-channel approach can effectively influence health-seeking behaviour when supported by political will. However, while increasing accessibility is crucial, participants also stressed that it is important to avoid inappropriate health-seeking behaviours:

*“By making ourselves excessively available, we're actually enabling bad health-seeking behaviour. We should be trying to encourage good health-seeking behaviour” (Interview 7, Male GP, 60+)​.*

### Major theme 3—Practice-level response

Participants widely acknowledged that to meet the demands of an evolving healthcare landscape, their own practices and approaches to medicine must adapt. Many highlighted general practice's long-standing adaptability, noting their early adoption of digital tools like computerization and electronic health records, as well as their pivotal role in the rapid rollout of COVID-19 vaccinations.

This theme explores how practices can evolve further to address current and future challenges, particularly through the Optimization of Technology and Reallocation of Roles. Together, these subthemes reflect the need for efficiency and flexibility in adapting to the ongoing shifts in patient needs and healthcare resources.

#### Optimization of technology

Participants viewed technology optimization as a crucial way to alleviate administrative burdens and allow GPs to focus on clinical care. Many expressed a desire to minimize routine tasks through digital solutions:

*“If we could minimize the administrative side as much as possible to allow [GPs] to concentrate on the medical work” (Interview 6, Male GP, 60+)​*.

GPs saw potential in advanced IT systems and artificial intelligence (AI) to streamline operations and improve efficiency, yet they were cautious about maintaining the personal connection with patients amidst these changes. The integration of AI was seen as particularly promising for promoting evidence-based care.

However, there were concerns that without sufficient training and support, new technologies might create more challenges than they resolve. The balance between technological efficiency and maintaining the human touch in patient interactions remains essential, with participants emphasizing that technology should support rather than replace the personal aspects of care.

#### Reallocation of roles

Participants highlighted that successful role reallocation within general practice requires time, clear communication, and cultural adaptation. While expanding support roles and delegating tasks can reduce GP workloads and improve service delivery, it can also alter practice dynamics and must be managed carefully.

Challenges to this approach include patient preferences to see a doctor, GPs' hesitance to relinquish certain responsibilities, and support staff's reluctance to assume new tasks. Participants also cautioned against excessive delegation, which could lead to de-skilling among GPs. Overcoming these challenges requires establishing strong governance structures, open communication, and comprehensive training to support role transitions and build trust:

*“Restructuring is essential within each GP practice. The critical question is who performs which tasks. With many GPs undertaking tasks better suited for nursing or administrative staff, and nurses performing tasks that could be delegated to phlebotomists or healthcare assistants, it's about ensuring the right person is doing the right job at the right time. The goal is for each practice to achieve an optimal workflow” (Interview 9, Male GP, 40–49)​*.

The strategies outlined in Table 2 are extrapolated from these themes using both macro- and micro-level solutions. At the macro-level, proactive government engagement and substantial infrastructure investment are emphasized, including purpose-built medical centres, improved public health communication, and strengthened connections between general practices and hospitals. At the micro-level, the focus is on practical, immediate changes, such as optimizing IT systems and redistributing administrative tasks to reduce GP workload and improve patient care. Collectively, these strategies provide a cohesive approach to tackling high-level policy issues and operational challenges within primary care.

**Table 2.** Synthesis of macro- and micro-level strategies for addressing GP workforce challenges.

Change type	Strategy	Solution
Macro	Proactive Advocacy and Government Engagement	Strengthening advocacy by organizations like the Irish College of General Practitioners and Irish Medical Organisation to influence healthcare policies effectively
	Enhancing Infrastructure	Government investment in building purpose-designed medical centres to reduce the financial burden on GPs
	Reliable Locum Systems	Creating a government-supported locum pool to ensure GPs can take necessary leave without compromising care
	Out-of-Hours Services Overhaul	Reforming out-of-hours services to differentiate routine from urgent care needs, funded by the Health Service Executive (HSE)
	Public Health Messaging	Launching public health campaigns to enhance health literacy and manage patient expectations
	Strengthening Interface Between General Practice and Hospitals	Improving referral processes and sharing patient records for seamless transitions between primary and secondary care
Micro	Enhancing Infrastructure	Providing flexible work arrangements, such as trial periods for new GPs, to find suitable work environments
	Optimizing Information Technology (IT) in General Practice	Implementing advanced IT systems like Health Link and Health Mail to reduce administrative workload
	Streamlining Workload and Administration	Delegating appropriate tasks to nursing, paramedics, phlebotomist and administrative staff to allow GPs to focus on clinical care
	Enhancing and Tailoring Primary Care Teams	Customizing primary care teams to meet the specific needs of local populations for more effective care

This table outlines both macro-level strategies and micro-level solutions aimed at supporting GPs and improving the sustainability of general practice in Ireland.

## Discussion

The aim of this study was to explore GPs' perspectives and identify key strategies to address Ireland's general practice workforce crisis. The qualitative analysis produces three important novel findings. Firstly, to encourage GPs to enter or continue a career in general practice, a whole-system approach, including systemic reforms in the entire healthcare system, investment in infrastructure, and rural healthcare resources, is needed. The second novel finding was that participants believe that engaging GPs in regional and national healthcare planning relating to the area in which they are working will ensure solutions align with local needs. Thirdly, structured support, such as role reallocation and enhanced digital tools, is essential for GPs to adapt effectively to evolving care demands.

Comprehensive structural reform is essential to address systemic shortcomings in Ireland's healthcare system, which participants identified as a major driver of GP dissatisfaction and workforce attrition. Inefficiencies and poor communication between primary and secondary care compromise patient safety and exacerbate GP workloads [29–31]. This aligns with Organisation for Economic Co-operation and Development (OECD), HSE, ICGP, and UK reports emphasizing sustained investment and rural workforce support [5, 15, 32, 33]. These findings also reflect evidence from the ESRI report showing how unmet healthcare needs and affordability barriers disproportionately affect patients in Ireland's mixed public–private system [34]. Without targeted investments in infrastructure and recruitment, the strain on GPs will intensify, particularly in underserved areas [5, 15, 31]. Measures such as purpose-built facilities, improved data-sharing systems, and recognizing GPs as key stakeholders in planning are critical, echoing the World Health Organisation's (WHO) call for primary care professional involvement to enhance sustainability [35].

Centralized locum support, targeted rural healthcare funding, and digital infrastructure are also vital to relieving pressures and attracting new GP entrants, particularly in underserved areas, as advocated by the ICGP and ESRI reports [3, 15, 34]. The OECD report recommends tailored rural investment as a key strategy for improving access and workforce retention [32]. The UK's National Health Service (NHS) experience, supported by the Health and Social Care Committee report, shows that centralized recruitment, community-based solutions, and rural funding can help alleviate pressures on general practice and improve access to care [5].

Clarifying roles within multidisciplinary teams is crucial for building a sustainable primary care model [6, 17, 36]. Reallocating routine tasks to allied health professionals, such as nurse practitioners and pharmacists, allows GPs to focus on complex cases, thereby enhancing care efficiency and quality [37]. Irish data similarly suggest the need for significant expansion of community-based nursing roles, with the ESRI recommending a 53% increase in such posts [33, 34]. This aligns with WHO's recommendation to optimize skill mix by redefining team structures to improve service quality [35]. The ICGP supports GP-led team models, which are in line with international evidence showing that role clarity, training, and governance are essential for effective integration [3, 5, 15, 32, 37–39]. For example, the UK's Primary Care Networks (PCNs) illustrate how redistributing responsibilities within teams can improve capacity and efficiency [5]. However, without clear guidelines and adequate governance, role ambiguity may lead to challenges in team cohesion, as observed in the UK experience [5, 17]. This model, when supported by structured training and clear role definitions, offers a compelling solution for Irish policymakers.

Digital infrastructure was consistently identified as central to the future of general practice. Practice-level digital adaptations, including IT optimization and administrative role reallocation, are essential for managing patient volumes and enhancing continuity of care. Digital systems like Ireland's HealthLink and HealthMail have shown potential in reducing administrative tasks and supporting patient-centred care, reflecting strategies from the Department of Health, Health Service Executive (HSE), and ESRI's emphasis on reforming and expanding digital infrastructure to address inefficiencies [33, 34, 40–42]. This perspective also aligns with the WHO Framework for Action and the Bucharest Declaration, which advocate for expanding digital infrastructure to increase efficiency and accessibility, particularly in under-resourced areas [35, 43]. Tailoring digital tools to local healthcare needs, as recommended by both UK and OECD reports, is critical to sustainable implementation [5, 32]. Ensuring these tools support, rather than substitute, direct patient care will be essential for effective integration. Evidence also shows that when resourced and embedded effectively, digital tools can increase patient uptake and reduce administrative burden [44].

### Future directions and research recommendations

Future research should focus on the long-term impacts of interventions, particularly the effectiveness of digital tools in rural settings and the scalability of role reallocation. Comparative studies with other European countries facing similar workforce shortages could provide further insights and support targeted policy refinements for Ireland's primary care system.

## Strengths and limitations

Strengths include the use of the ULEARN network, which enabled diverse Irish GP perspectives, including from different ages, genders, geographic locations, and practice settings—a broad range of experiences and insights relevant to the general practice workforce in Ireland. The rigorous thematic analysis approach further strengthens the study, with data collection from a sample far exceeding the minimum accepted for thematic analysis by its creators and achieving thematic saturation [27].

While participation of the study was limited to GPs, and this may be seen as a limitation, this decision was taken following an initial pilot focus group with GPs during which it was evident that the voice of GPs needed to be heard. Future research should incorporate GPs, other practice staff, and patients. Potential self-selection bias was identified by the researchers as a concern, as GPs facing more acute challenges could have been more likely to participate. However, the nature of this present qualitative research sought not to establish themes that were statistically true for all GPs but rather to explore the nature and depth of the challenges faced in general practice by those who participated, who expressed their concerns in a reasonable and sophisticated manner. Nevertheless, there is potential to extend this research further and to examine a larger, randomized, and national sample of GPs in future studies.

## Conclusion

GPs will be attracted to work and remain in Ireland when the wider health system is significantly improved. The process of improving the health system must include general practitioners at national, regional, and local levels.

## Supplementary Material

cmaf094_Supplementary_Data

## Data Availability

The authors will make data available on reasonable request.

## References

[1] Svedahl ER, Pape K, Toch-Marquardt M et al Increasing workload in Norwegian general practice—a qualitative study. BMC Fam Pract 2019;20:68. 10.1186/s12875-019-0952-5 31113368 PMC6530128

[2] Schafer WLA, van den Berg MJ, Groenewegen PP. The association between the workload of general practitioners and patient experiences with care: results of a cross-sectional study in 33 countries. Hum Resour Health 2020;18:76. 10.1186/s12960-020-00520-9 33066776 PMC7565810

[3] (ICGP) Irish College of General Practitioners . *Shaping the Future: A Discussion Paper on the Workforce & Workload Crisis in General Practice in Ireland*. 2022:50. https://www.irishcollegeofgps.ie/Portals/0/Clinical Hub/Publications and Journals/Current%20Publications/CH_Pub_Current_Shaping_the_Future_October_2022.pdf?ver=1EmY9yRIyeedqPgNVMd9mw%3d%3d (19 September 2025, date last accessed).

[4] Marshall M . The future of general practice in England. BMJ 2022;379:o2554. 10.1136/bmj.o2554

[5] Health and Social Care Committee . *The Future of General Practice*. Health and Social Care Committee, 2022. https://publications.parliament.uk/pa/cm5803/cmselect/cmhealth/113/report.html (19 September 2025, date last accessed).

[6] Owen K, Hopkins T, Shortland T et al GP retention in the UK: a worsening crisis. Findings from a cross-sectional survey. BMJ open 2019;9:e026048. 10.1136/bmjopen-2018-026048 PMC6398901 30814114

[7] Collins C, Homeniuk R. How many general practice consultations occur in Ireland annually? Cross-sectional data from a survey of general practices. BMC Fam Pract 2021;22:40. 10.1186/s12875-021-01377-0 33610171 PMC7896162

[8] Health Service Executive (HSE) . *National Doctors Training & Planning (NDTP) 2020 v.2. Demand for Medical Consultants and Specialists to 2028 and the Training Pipeline to Meet Demand: A High Level Stake Holder Informed Analysis*. Health Service Executive, 2020. https://www.hse.ie/eng/staff/leadership-education-development/met/plan/demand-for-medical-consultants-and-specialists-to-2028-november-updates-v2.pdf (19 September 2025, date last accessed).

[9] Crosbie B, O'Callaghan ME, O'Flanagan S et al A real-time measurement of general practice workload in the Republic of Ireland: a prospective study. Br J Gen Pract 2020;70:e489–96. 10.3399/bjgp20X710429 32482628 PMC7274543

[10] Connolly S, Kakoulidou T, McHugh E. *Projections of National Demand and Workforce requirements for general practice in Ireland, 2023–2040: Based on the Hippocrates model*. 2025. https://www.esri.ie/publications/projections-of-national-demand-and-workforce-requirements-for-general-practice-in (19 September 2025, date last accessed).

[11] Irish College of GPs . *Strengthening the Future of GP Care in Ireland*. Vol. 19/9/2025, 11:26:05. July 2025:1.0. https://www.irishcollegeofgps.ie/Portals/0/Explore the College/About Us/Advocacy/Submissions/Strengthening the future 2025.pdf?ver=Io5WdKlHsUVHZjRDDjUbMA== (19 September 2025, date last accessed).

[12] Hanlon HR, Shé ÉN, Byrne J-P et al GP emigration from Ireland: an analysis of data from key destination countries. BMC Health Serv Res 2024;24:1628. 10.1186/s12913-024-12117-2 39707341 PMC11660595

[13] Hall LH, Johnson J, Watt I et al Association of GP wellbeing and burnout with patient safety in UK primary care: a cross-sectional survey. Br J Gen Pract 2019;69:e507. 10.3399/bjgp19X702713 31015224 PMC6592321

[14] Harbitz MB, Stensland PS, Gaski M. Rural general practice staff experiences of patient safety incidents and low quality of care in Norway: an interview study. Fam Pract 2021;39:130–6. 10.1093/fampra/cmab064 PMC8826993 34180505

[15] The Irish College of General Practitioners . *Submission to the Oireachtas Joint Committee on Health*. 2022. https://www.irishcollegeofgps.ie/Portals/0/Clinical Hub/Publications and Journals/Current Publications/CH_Pub_Current_Submission_to_the_Oireachtas_Joint_Committee_on_Health_2022.pdf (19 September 2025, date last accessed).

[16] Long L, Moore D, Robinson S et al Understanding why primary care doctors leave direct patient care: a systematic review of qualitative research. BMJ Open 2020;10:e029846. 10.1136/bmjopen-2019-029846 PMC7228506 32404383

[17] Fisher RF, Croxson CH, Ashdown HF et al GP views on strategies to cope with increasing workload: a qualitative interview study. Br J Gen Pract 2017;67:e148–56. 10.3399/bjgp17X688861 28093421 PMC5308121

[18] Marchand C, Peckham S. Addressing the crisis of GP recruitment and retention: a systematic review. Br J Gen Pract 2017;67:e227–37. 10.3399/bjgp17X689929 28289014 PMC5565821

[19] Tandan M, Twomey B, Twomey L et al National chronic disease management programmes in Irish general practice-preparedness and challenges. J Pers Med 2022;12:1157. 10.3390/jpm12071157 35887654 PMC9323818

[20] Statistics & Analytics Services Department of Health . *Health in Ireland Key Trends 2023*. Health Do, 2024. https://assets.gov.ie/static/documents/health-in-ireland-key-trends-2023.pdf (19 September 2025, date last accessed).

[21] Central Statistics Office . *Health Care Ireland's UN SDGs—Goal 3 Good Health and Well-Being 2024*. Central Statistics Office, 2024. https://www.cso.ie/en/releasesandpublications/ep/p-sdg3/irelandsunsdgs-goal3goodhealthandwell-being2024/healthcare/ (19 September 2025, date last accessed).

[22] Mattsson M, Flood M, Wallace E et al Eligibility rates and representativeness of the General Medical Services scheme population in Ireland 2016–2021: a methodological report [version 2; peer review: 2 approved]. HRB Open Res 2023;5:67. 10.12688/hrbopenres.13622.2 37753170 PMC10518849

[23] Tong A, Sainsbury P, Craig J. Consolidated criteria for reporting qualitative research (COREQ): a 32-item checklist for interviews and focus groups. Int J Qual Health Care 2007;19:349–57. 10.1093/intqhc/mzm042 17872937

[24] O'Regan A, Hayes P, O'Connor R et al The University of Limerick Education and Research Network for General Practice (ULEARN-GP): practice characteristics and general practitioner perspectives. BMC Fam Pract 2020;21:25. 10.1186/s12875-020-1100-y 32024480 PMC7003418

[25] Braun V, Clarke V. Using thematic analysis in psychology. Qual Res Psychol 2006;3:77–101. 10.1191/1478088706qp063oa

[26] Braun V, Clarke V. Successful Qualitative Research: A Practical Guide for Beginners. SAGE Publications Ltd, London: SAGE Publications, 2013. https://us.sagepub.com/en-us/nam/successful-qualitative-research/book233059.

[27] Braun V, Clarke V. (Mis)conceptualising themes, thematic analysis, and other problems with Fugard and Potts’ (2015) sample-size tool for thematic analysis. Int J Soc Res Methodol 2016;19:739–43. 10.1080/13645579.2016.1195588

[28] Berger R . Now I see it, now I don’t: researcher's position and reflexivity in qualitative research. Qual Res 2015;15:219–34. 10.1177/1468794112468475

[29] Dinsdale E, Hannigan A, O’Connor R et al Communication between primary and secondary care: deficits and danger. Fam Pract 2020;37:63–8. 10.1093/fampra/cmz037 31372649

[30] Madden C, Lydon S, Murphy AW et al Patients’ perception of safety climate in Irish general practice: a cross-sectional study. BMC Fam Pract 2021;22:257. 10.1186/s12875-021-01603-9 34961484 PMC8710927

[31] Fattahi H, Seproo FG, Fattahi A et al General Practitioners’ perspectives on barriers to communication with specialists in the referral system: a systematic review and meta-synthesis. Health Sci Rep 2025;8:e70785. 10.1002/hsr2.70785 40415981 PMC12098960

[32] OECD . *Health at A Glance 2023*. 2023. https://www.oecd-ilibrary.org/content/publication/7a7afb35-en (11 November 2023, date last accessed).

[33] O’Dowd T, Ivers J-H, Handy D. *A Future Together: Building a Better GP and Primary Care Service*. 2017. https://www.hse.ie/eng/services/publications/primary/a-future-together.pdf (19 September 2025, date last accessed).

[34] Connolly S, Wren M-A, Keegan C, et al Universal Primary Care in Ireland: cost and workforce implications. Econ Soc Rev (Irel) 2022;53:281–98. https://www.esri.ie/system/files/publications/JA202258.pdf (19 September 2025, date last accessed).

[35] World Health Organization. Regional Office for Europe . *World Health Organization Regional Office for Europe (WHO/Europe). Framework for action on the health and care workforce in the WHO European Region 2023–2030*. Copenhagen: WHO/Europe, 2023. https://www.who.int/europe/publications/i/item/EUR-RC73-8 (19 September 2025, date last accessed).

[36] Torrens C, Campbell P, Hoskins G et al Barriers and facilitators to the implementation of the advanced nurse practitioner role in primary care settings: a scoping review. Int J Nurs Stud 2020;104:103443. 10.1016/j.ijnurstu.2019.103443 32120089

[37] Groenewegen P, Boerma WGW, Spreeuwenberg P et al Task shifting from general practitioners to practice assistants and nurses in primary care: a cross-sectional survey in 34 countries. Prim Health Care Res Dev 2022;23:e60. 10.1017/S1463423622000470 36134523 PMC9532851

[38] Lewis R . Developing a ‘national module’ for nurses considering a career in general practice: addressing the workforce crisis in primary care. Practice Nursing 2024;35:136–9. 10.12968/pnur.2024.35.4.136

[39] Casey M, O'Connor L, Rohde D et al Role dimensions of practice nurses and interest in introducing advanced nurse practitioners in general practice in Ireland. Health Sci Rep 2022;5:e555. 10.1002/hsr2.555 35284651 PMC8905424

[40] eHealth Ireland . *Healthlink*. https://www.ehealthireland.i.e./technology-and-transformation-functions/access-to-information-a2i/healthlink1/files/635/healthlink1.html (19 September 2025, date last accessed).

[41] Health Service Executive (HSE) . *Healthmail Registration Portal*. https://www.healthmail.i.e./index.cfm (19 September 2025, date last accessed).

[42] Health Service Executive (HSE) . *Digital Roadmap: Transforming the Online User Experience for Health*. 2017. https://www.hse.ie/eng/about/who/communications/digital/digital-transformation/hse-digital-roadmap-web.pdf (19 September 2025, date last accessed)

[43] World Health Organization; Regional Office for Europe; World Health Organization Regional Office for Europe (WHO/Europe) . *Bucharest Declaration on Health and Care Workforce*. Copenhagen: WHO/Europe, 2023. https://www.who.int/europe/publications/i/item/bucharest-declaration (19 September 2025, date last accessed).

[44] Sussex J, Atherton H, Abel G et al Supporting patients’ use of digital services in Primary Health Care in England: synthesis of evidence from a mixed methods study of “Digital Facilitation”. JMIR Hum Factors 2024;11:e52516. 10.2196/52516 39630414 PMC11633515

