# Supplementary material for: Addressing systemic workforce challenges in general practice—a qualitative study of general practitioners in Ireland
Source: Fam Pract. 2025 Dec 9;43(1):cmaf094. doi: 10.1093/fampra/cmaf094 (PMC12686944; doi:10.1093/fampra/cmaf094)
Supplement: cmaf094_Supplementary_Data [file cmaf094_supplementary_data.pdf]

Addressing systemic workforce challenges in general practice – a qualitative study of general practitioners in Ireland

Consolidated criteria for reporting qualitative studies (COREQ): 32-item Checklist[1]

Domain 1: Research Team and Reflexivity

No.	Item	Guide Questions/Description	Answer
**Personal Characteristics**			
1	Interviewer/facilitator	Which author/s conducted the interview or focus group?	The interviews were conducted by first author (Uzair Shabbir), a practising GP.
2	Credentials	What were the researcher's credentials? E.g., PhD, MD	(Details of all authors are on Page 1) First author, MD,MICGP, Cert in Lifestyle Medicine, Fellowship in Physical Activity for Chronic Disease.
3	Occupation	What was their occupation at the time of the study?	Practising GP and research assistant.
4	Gender	Was the researcher male or female?	Male.
5	Experience and training	What experience or training did the researcher have?	Experienced GP and researcher with a professional interest in healthcare system challenges.
**Relationship with Participants**			
6	Relationship established	Was a relationship established prior to study commencement?	A relationship was established through an invitation email, scheduling of interviews, and rapport-building at the start of each interview

No.	Item	Guide Questions/Description	Answer
7	Participant knowledge of interviewer	What did the participants know about the researcher? e.g., personal goals, reasons for study	Participants were informed of the researcher’s role and research goals. (Page 5)
8	Interviewer characteristics	What characteristics were reported about the interviewer?	GP with interest in workforce challenges.

Domain 2: Study Design

No.	Item	Guide Questions/Description	Answer
**Theoretical Framework**			
9	Methodological orientation and theory	What methodological orientation was stated to underpin the study? e.g., grounded theory	Inductive thematic analysis following Braun and Clarke's framework.[2](Page 6)
**Participant Selection**			
10	Sampling	How were participants selected? e.g., purposive, convenience, consecutive, snowball	Purposive sampling ensured diverse participant representation. (Page 5)
11	Method of approach	How were participants approached? e.g., face-to-face, telephone, mail, email	Participants were recruited via email.
12	Sample size	How many participants were in the study?	The study included 21 participants.(Table 1)
13	Non-participation	How many people refused to participate or dropped out? Reasons?	No information provided on non-participation or dropout.
**Setting**			

No.	Item	Guide Questions/Description	Answer
14	Setting of data collection	Where was the data collected? e.g., home, clinic, workplace	Interviews were conducted online using Microsoft Teams.
15	Presence of non-participants	Was anyone else present besides the participants and researchers?	Only participants and the interviewer were present.
16	Description of sample	What are the important characteristics of the sample? e.g., demographic data, date	The study included 21 Irish GPs, representing diverse age groups (30s to 60+), genders, and practice roles, including partners, salaried GPs, and trainers. Participants were drawn from both urban and rural settings to capture varied professional experiences and challenges.(Table 1)
**Data Collection**			
17	Interview guide	Were questions, prompts, guides provided by the authors? Was it pilot tested?	A semi-structured guide, informed by pilot testing, was iteratively refined during the study.
18	Repeat interviews	Were repeat interviews carried out? If yes, how many?	No repeat interviews were conducted.
19	Audio/visual recording	Did the research use audio or visual recording to collect the data?	Interviews were audio-recorded digitally.
20	Field notes	Were field notes made during and/or after the interview or focus group?	Field notes were taken during and after interviews.
21	Duration	What was the duration of the interviews or focus group?	Interviews lasted between 20–60 minutes.
22	Data saturation	Was data saturation discussed?	Yes. Thematic saturation was achieved, ensuring comprehensive representation of views.(Page 6)

No.	Item	Guide Questions/Description	Answer
23	Transcripts returned	Were transcripts returned to participants for comment and/or correction?	Transcripts were not returned to participants for review.

Domain 3: Analysis and Findings

No.	Item	Guide Questions/Description	Answer
**Data Analysis**			
24	Number of data coders	How many data coders coded the data?	Four researchers participated in data analysis.(Page 6)
25	Description of the coding tree	Did authors provide a description of the coding tree?	A formal coding tree was not developed; themes were categorised into conceptual groupings inductively.
26	Derivation of themes	Were themes identified in advance or derived from the data?	Themes were derived inductively through iterative team discussion from the data.
27	Software	What software, if applicable, was used to manage the data?	Microsoft word was used to manage the data.
28	Participant checking	Did participants provide feedback on the findings?	No
**Reporting**			
29	Quotations presented	Were participant quotations presented to illustrate the themes/findings?	Participant quotations were used to illustrate findings, with demographic identifiers included. (Results section page 7 -13)
30	Data and findings consistent	Was there consistency between the data presented and the findings?	Findings were consistent with data and supported by quotations.
31	Clarity of major themes	Were major themes clearly presented?	Major themes were clearly defined and supported by evidence. (Figure 1,Results section page 7 -13)
32	Clarity of minor themes	Is there a description of diverse cases or discussion of minor themes?	Minor themes were discussed with diverse cases highlighted. (Figure 1,Results section page 7 -13)

1. Tong A, Sainsbury P, Craig J. Consolidated criteria for reporting qualitative research (COREQ): a 32-item checklist for interviews and focus groups. *Int J Qual Health Care*. Dec 2007;19(6):349-57. doi:10.1093/intqhc/mzm042
2. Braun V, Clarke V. Using thematic analysis in psychology. *Qualitative Research in Psychology*. 2006/01/01 2006;3(2):77-101. doi:10.1191/1478088706qp063oa
